# Supplementary material for: Photonic orbital angular momentum with controllable orientation
Source: Natl Sci Rev. 2021 Aug 16;9(7):nwab149. doi: 10.1093/nsr/nwab149 (PMC9359470; doi:10.1093/nsr/nwab149)
Supplement: nwab149_Supplemental_File [file nwab149_supplemental_file.docx]

**­­Supplementary information:**

**Photonic orbital angular momentum with controllable orientation**

**I. Supplementary Method**

**

­­­­­­**

**Fig. S1. Experimental setup to generate and characterize the intersection of spatiotemporal vortices and spatial vortices.** The wave packet emitted from a mode-locked fiber laser acquires a spatiotemporal vortex through a pulse shaper and two-dimensional spatiotemporal Fourier transform. A spatial vortex is then applied to the wave packet by a second SLM. (Revised from Fig. 1a in Ref. [16])

The experimental setup starts with a mode-locked fiber laser that emits a chirped pulse of around 3ps with a central wavelength of 1030 nm (Fig. S1). A following pulse shaper that consists of a diffraction grating, a cylindrical lens and a two-dimensional spatial light modulator (SLM) applies a spiral phase in the spatial frequency - temporal frequency (*k*-ω) domain and performs a two-dimensional spatiotemporal Fourier transform. The generated wave packet with spatiotemporal vortices then acquires a spatial spiral phase modulation from another two-dimensional SLM. Consequently, the intersection of spatiotemporal vortices and spatial vortices is achieved within a chirped wave packet. A split pulse from the laser source is dechirped by a grating pair and utilized as a reference pulse. The reference pulse is shortened to around 90 fs. With the help of a precision linear stage, the reference pulse slices through the chirped wave packet and obtains slices of interference fringe patterns that characterize the three-dimensional optical field of the chirped wave packet. The details of the characterization technique can be found in [14].

**II. Supplementary Data**

Figure S2 shows the demonstration of a chirped wave packet that contains a spatiotemporal vortex of topological charge 1 with a spatial vortex of topological charge 1. Unlike the case shown in Fig. 2, the two vortex tunnels are not intersected. In Figs. S2a to S2d, the peripheral fringes display the feature of a spatiotemporal vortex of topological charge 1. The upper half of peripheral fringes first bend to the right in Fig. S2b, and then to the left in Fig. S2d after passing the $\pi$ phase shift position shown in Fig. S2c. The spatial vortex structure is not greatly influenced by the spatiotemporal vortex because the cores of the two vortices are not intersected. Therefore, a forklike pattern pointing downwards, the feature of a spatial vortex of topological charge 1, is constantly shown in all fringe patterns. Figures S2e and S2f show the three-dimensional intensity reconstruction of the chirped wave packet. Clearly, the two vortex tunnels are not intersected.





**Fig. S2. A chirped wave packet that contains a spatiotemporal vortex of topological charge 1 with a spatial vortex of topological charge 1.** The two vortex tunnels are not intersected. (a-d) Interference fringe patterns of the reference pulse with the chirped wave packet at various positions. Black dashed lines mark the bending directions of fringes. (e,f) Three-dimensional intensity reconstruction of the chirped wave packet from different views. 85.7% energy of the wave packet is contained in the isosurface. Temporal separation between slice 1,2,3 and 4 are 1ps, 83fs, 83fs, respectively.

Figures S3a to S3b show the demonstration of a chirped wave packet that contains a spatiotemporal vortex of topological charge 1 with a spatial vortex of topological charge 2. Using the same isovalue of 0.05, the spatial vortex of topological charge 2 shows a larger hole as shown in Fig. S3b when comparing with the case shown in Fig. 2. Figures S3c to S3d show the demonstration of a chirped wave packet that contains a spatiotemporal vortex of topological charge -1 with a spatial vortex of topological charge -2. The twisting direction of both vortex tunnels are switched because the signs of the topological charge are flipped.


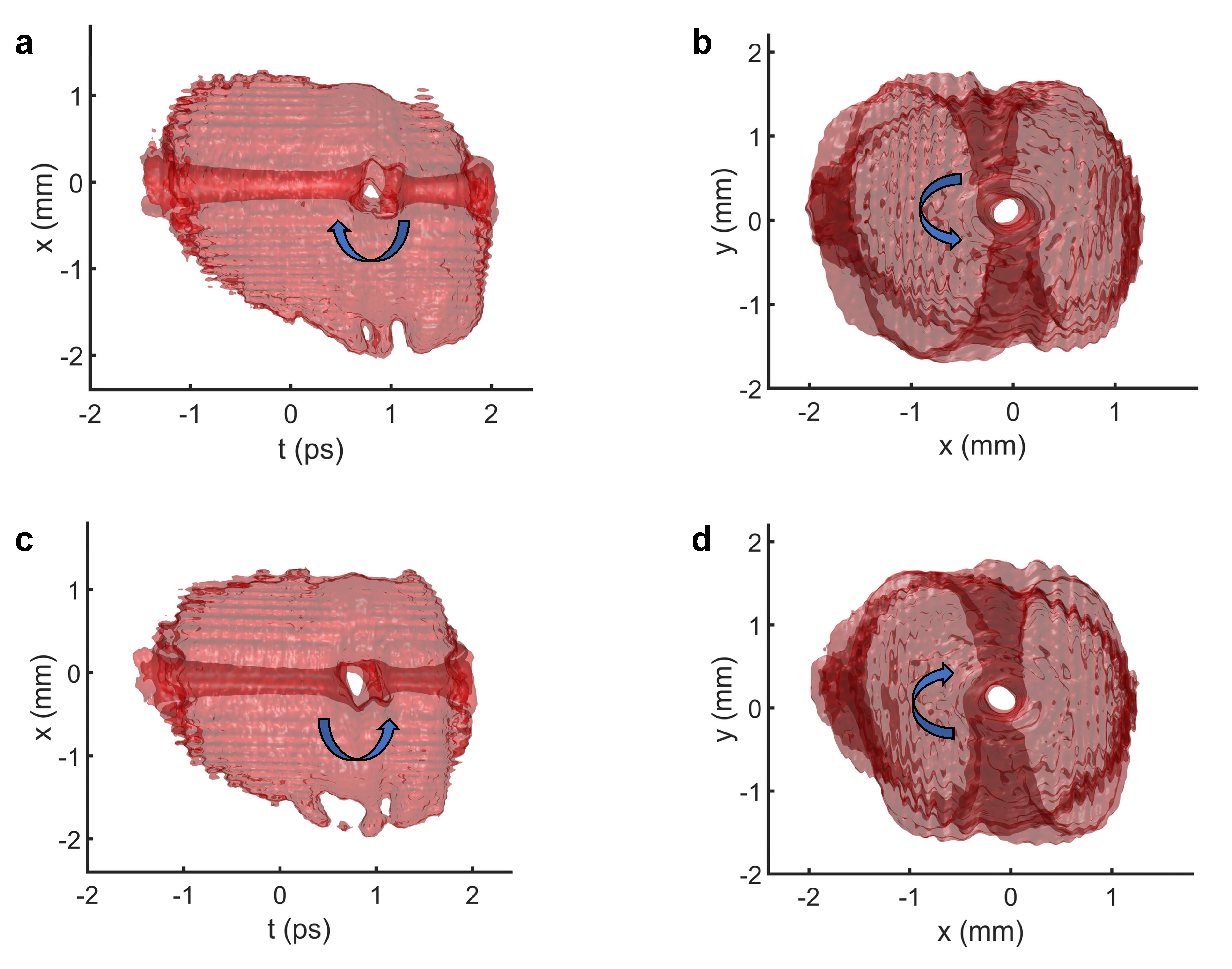


**Fig. S3. A chirped wave packet that contains a unitary spatiotemporal vortex and a high-order spatial vortex. (a,b)** The topological charge of the spatiotemporal vortex and spatial vortex are 1 and 2, respectively. 85.5% energy of the wave packet is contained in the isosurface. (c,d) The topological charge of the spatiotemporal vortex and spatial vortex are -1 and -2, respectively. 83.4% energy of the wave packet is contained in the isosurface.
